# Bio-C (Modified Hyaluronic Acid-Coated-Collagen Tube) Implants Enable Functional Recovery after Complete Spinal Cord Injury

**DOI:** 10.3390/pharmaceutics14030596

**Published:** 2022-03-09

**Authors:** Changhong Zheng, Huina Zhang, Yanling Cui, Yuchen Mu, Kun Jiang, Liqiang Zhou, Junbang Wang, Jiping Liu, Yaxuan Deng, Chunxue Zhang, Wenmin Zhu, Kongyan Wu, Yi Eve Sun

**Affiliations:** 1 Stem Cell Translational Research Center, Tongji Hospital, School of Medicine, Tongji University, Shanghai 200065, China; huinazhang0@163.com (H.Z.); cuiyanling0806@163.com (Y.C.); 1653118@tongji.edu.cn (Y.M.); jiangk1994@163.com (K.J.); 2013jasonzhou@tongji.edu.cn (L.Z.); wjbang520@163.com (J.W.); zacharylau10@hotmail.com (J.L.); d769971156@163.com (Y.D.); zhangchunxue91@163.com (C.Z.); zwm8899@163.com (W.Z.); wukongyan@live.com (K.W.); 2 Shanghai Institute of Stem Cell Research and Clinical Translation, Shanghai East Hospital, School of Medicine, Tongji University, Shanghai 200120, China

**Keywords:** Bio-C, spinal cord injury (SCI), neurotrophic, anti-inflammatory, ADC

## Abstract

Neural repair within the central nervous system (CNS) has been extremely challenging due to limited abilities of adult CNS neurons to regenerate, particularly in a highly inflammatory injury environment that is also filled with myelin debris. Spinal cord injury (SCI) is a serious medical condition that often leads to paralysis and currently has no effective treatment. Here we report the construction of a novel biocompatible and biodegradable material, Bio-C, through coating of acid-desalted-collagen (ADC) tube with pre-modified hyaluronic acid, which, after implantation, can elicit quite robust neural regeneration and functional recovery after complete spinal-cord transection with a 2 mm–spinal-cord-segment removal in mice. We combined morphological, electrophysiological, and objective transcriptomic analyses, in addition to behavioral analyses, to demonstrate neural tissue regeneration and functional recovery through the establishment of Bio-C-induced anti-inflammatory, neurogenic, and neurotrophic microenvironment. Through this study, we unveiled the underlying logic for CNS neural repair.

## 1. Introduction

A spinal cord injury (SCI) is generally considered to be incurable, because it is well-known that neurons in the central nervous system (CNS), in contrast to those in the peripheral nervous system (PNS), do not possess strong axonal regeneration properties [1]. In addition, CNS neural regeneration is also hampered by a harmful inflammatory microenvironment and lots of myelin debris, which are inhibitory for axonal growth [2]. SCI is always accompanied by the breakage of blood vessels and axons, death of neurons, immune cell infiltration, and inflammation. In the past, the SCI repair field mainly focused on the eradication of inhibitory factors for axonal regeneration and/or enhancement of innate axonal regeneration capacity of CNS neurons [2,3], but with limited success. In recent years, new strategies involving the establishment of anti-inflammatory and neural trophic environment to enable the plasticity of spared neurons to remodel local relay neural networks to allow functional recovery after SCI [4,5] appeared to be more applicable or translatable in clinics. Moreover, in the case of complete SCI with spinal-cord-tissue removal, nascent relay neural networks could also be established via de novo genesis of new neurons from endogenous neural stem cells (NSCs) [6,7]. Such relay or patching neural networks can be integrated into damaged functional neural circuits to resume function [6,8]. These new strategies do not require long-distance axonal regeneration or pathfinding; however, the quiet extensive axonal growth from spared and/or newly generated neurons concomitantly occurs [6,7,9].

When a permissive and regenerative microenvironment is provided, much better functional recovery becomes possible after SCI [10,11,12,13]. Previously, our own study demonstrated that better functional recovery after SCI occurred in immunocompromised SCID mice as compared to C57/black6 wild-type mice, due to the heightened neural connectivity in SCID mice [4]. The application of degradable bioactive materials could be a feasible strategy to build a beneficial microenvironment for neural repair after SCI. Some natural materials, such as chitosan, collagen, and hyaluronic acid, exhibit excellent biocompatibility, biodegradability, and can easily be fabricated; some of them have already been applied in SCI animal models and demonstrated promising results [6,14,15]. Our previous work reported that neutrophin-3 (NT-3)-loaded chitosan provided a great microenvironment, which was anti-inflammatory, neurogenic, neurotrophic, and angiogenic, for SCI repair and functional recovery in SCI rat and monkey models [6,7]. The formulation of the bioactive material allowed for the slow release of the potent neural trophic factor, NT-3, at physiological conditions for over a period of 14 weeks [16]. However, whether chitosan is already the best polymer to carry NT-3 remains an open question.

Collagen, with triple helix structures, has been proven to possess good elasticity, flexibility, and strength. It is produced by cells in the body as a major extracellular matrix (ECM) protein, which can promote cell growth, and is bioactive with excellent compatibility for tissue repair [14,17]. Following a slight wash with hydrogen chloride (HCl) and desalting through dialysis, collagen could be separated from bovine fascia and maintain the triple-helix structural integrity as fibrils, referred to as ADC (acid-desalt-collagen). Hyaluronic acid (HA), a highly biocompatible ECM component, has demonstrated efficacies in reducing scar formation, modulating neuroinflammation, and enhancing neural regeneration in CNS [18,19], especially with high molecular weight (HMW) [19]. The structural and morphological features of collagen-based materials, including porosity and fiber density, may influence the mechanical behavior and performance of material [20]. The structures and pore distributions of collagen-based scaffold can be modified by hyaluronic acid to increase cell viability and proliferation [21]. Considering implant structural construction, we postulated that, through the “freeze-dry” processes, we could combine ADC and modified HA to fabricate uniaxial and relatively even porous scaffolds to offer physical guidance for cell migration, as well as axonal outgrowth [13,22]. The aim of this study was to create a novel biodegradable material, which has good biocompatibility, and to evaluate its properties and efficacies in spinal-cord-injury repair.

In this study, combining collagen fibril ADC with HMW HA, we invented a new biodegradable material, i.e., a modified hyaluronic acid–coated-ADC tube called “Bio-C”, through the “freeze-drying” process. The biological effect of Bio-C was evaluated on the regulation of NSC activities in vitro, stimulation of neural regeneration, immune regulation, and functional recovery after SCI in vivo. Bio-C-treated animals revealed better functional recovery after SCI, compared to the no-treatment control and ADC groups, as measured by Basso Mouse Scale (BMS) scores, motor evoked potential (MEP), and immunohistochemical analyses of the spinal-cord tissues with neuronal markers and microglial markers. In NSC culture, Bio-C decreased NSC apoptosis and promoted neuronal differentiation. Transcriptomic analyses objectively revealed potential mechanisms for Bio-C to modulate neural regeneration after SCI. In summary, this study revealed that Bio-C, HA-coated collagen fibril, provided a beneficial microenvironment for neural regeneration after SCI. Given the molecular nature of Bio-C, it should have great translational potentials to be used in the clinic for spinal-cord-injury repair in the future.

## 2. Materials and Methods

### 2.1. Preparations of Bio-C

Type I collagen from bovine fascia was separated and washed with HCl (0.01 M) and dialyzed in sterile deionized water until the pH reached 7.2, after three rounds of water changes, and then dried. A 500 mg high-molecular-weight (HMW) sodium hyaluronate (viscosity 25.5–36.6 dL/g) purchased from Kewpie Corporation was dissolved in 2 mL of sterile deionized water at pH 7.2 and allowed to swell for about 24 h at 4 °C. Another portion of the HMW sodium hyaluronate was crosslinked by 1,4-butanediol diglycidal ether (BDDE) to produce modified hyaluronic acid (mHA) based on a previously developed method (Patent #: CN 201410154316). The mHA was then mixed with hydrated HMW sodium hyaluronate at a ratio of 1:10 and allowed for further swell for about 24 h, at 4 °C, before being mixed with collagen. The HA–collagen mix was frozen and vacuum-dried, followed by mixing with hydrated sodium hyaluronate solution. This final mixture was refrozen and vacuum-dried, and it was eventually stored at 4 °C for use.

*Reagents:* T1,4-butanediol-diglycidyl-ether (BDDE, Sigma-Aldrich 220892, Darmstadt, Germany), hydrochloric acid (HCl; 10011018), Tris-HCl (T1150), and sodium dodecyl sulfonate (SDS; S1010) were obtained from Solarbio, Beijing, China.

### 2.2. Characterization of Bio-C and Morphological Examination

Field-emission scanning electron microscopy (FEI Quanta 200, Hillsboro, OR, USA) was used to observe Bio-C and Bio-C combined with spinal-cord tissue after implantation. The morphology and structures of the scaffolds were analyzed. Specimens were dehydrated though a graded series of sucrose solutions and a “freeze-drying” cycle, and then sputter-coated with an ultrathin layer of gold. The average diameter of the pores was analyzed in an arbitrary zone, using a computerized image analyzer.

A transmission electron microscope (TEM, JEOL-1230, Akishima City, Tokyo, Japan) was used to analyze the morphology of Bio-C. The samples were cut and dispersed in double-distilled water, and several drops of each suspension were placed on a carbon-coated copper TEM grid.

### 2.3. Animals

Six-to-eight-week-old female mice (C57BL/6) at 18–20 g body weight were used; they were purchased from Beijing Vital River Laboratory Animal Technology (Beijing, China). All animals were raised in a specific pathogen-free (SPF) facility, under a 12 light/dark cycle, and had ad libitum access to food and water. Animals that failed to reach the endpoint of the experiment were excluded. All animals were randomly grouped by investigators. The group allocation, behavioral test, and assessment of outcome were performed by different investigators in a double-blinded manner. After surgery, animals were returned to their home cages and received manual bladder expression once daily. Animals were monitored every day for potential infections, abnormal wound healing, or unintentional weight loss. All experimental procedures complied with international guidelines for the care and use of laboratory animals and were approved by the Animal Ethics Committee of Tongji University, Shanghai, China (Approval Number: TJAA06621105, 10 February 2021 of approval).

### 2.4. Transection SCI Model (Spinal-Cord Surgery and Bio-C Implantation)

Surgeries were conducted under deep anesthesia induced by isoflurane. Firstly, laminectomy was performed to expose the dorsal surface of the T8-T10 spinal-cord segments. Transection was then introduced at the right side of the spinal cord by cutting twice at the same place, using fine ophthalmic scissors. Following laminectomy at the T9 vertebral level, the spinal cord was transected and a 2.0 mm cord segment, including visible spinal roots, was completely removed at the T9 spinal-cord level. The rostral and caudal stumps of the spinal cord retracted, and a 3.0 mm gap formed. Any residual fibers at the lesion site were removed under a microscope, with visual verification to ensure complete transection ventrally and laterally. After hemostasis was achieved, Bio-C grafts were rinsed in sterile normal saline and then placed between the rostral and caudal stumps. At the end of surgery, muscles and skin were sutured separately. Finally, animals were allowed to recover on a heating pad.

### 2.5. Tissue Processing

After excessive inhalation of isoflurane, animals were transcardially perfused with 4% paraformaldehyde (PFA, Sigma, Darmstadt, Germany) in phosphate buffer saline (PBS, pH 7.4, Sigma, Darmstadt, Germany). The spinal cord was removed and placed in 4% PFA, at 4 °C, overnight; then sequentially transferred first to 10% sucrose at 4 °C, overnight; and then 30% sucrose (Sigma, Darmstadt, Germany) overnight, at 4 °C. Samples were photographed under a microscope (Nikon, Tokyo, Japan), followed by tissue embedding in OCT compound (Sakura, Tokyo, Japan). A 1 cm spinal-cord segment centered on the lesion core was encapsulated in OCT at −80 °C. Tissues were cryo-sectioned at a thickness of 20 μm, using a frozen slicer (Leica, Nussloch, Germany), and mounted on charged glass slides. The sections were stained by hematoxylin–eosin (HE, Sigma, Darmstadt, Germany) to observe histological structures of tissues after SCI.

### 2.6. Immunohistochemistry

Sections were washed three times with 1× PBS and then incubated with primary antibodies at 4 °C overnight, after 1 h blocking by 5% normal goat serum (NGS, Sigma, Darmstadt, Germany) and 0.2% Triton X-100 (Sigma, Darmstadt, Germany). The sections were then incubated at room temperature for 2 h with fluorescence conjugated secondary antibodies (Invitrogen, Waltham, MA, USA) and washed with 0.01 M PBS (1×) 3 times before being observed under a confocal laser scanning microscope (Zeiss, LSM800, Jena, Germany). Fluorescence immunohistochemistry was performed by using the following primary antibodies: mouse anti-NF160 (Abcam, 1:400, Cambridge, UK), rabbit anti-NeuN (Abcam, 1:500, Cambridge, UK), chicken anti-Map2 (Abcam, 1:500, Cambridge, UK), rabbit anti-Gfap (Dako, 1:1000, Carpinteria, CA, USA), rabbit anti-Sox2 (Abcam, 1:200, Cambridge, UK), chicken anti-Gfap (Abcam, 1:1000, Cambridge, UK), guinea-pig anti-Iba1 (Synaptic Systems, 1:800, Göttingen, Germany), mouse anti-Tuj1 (R&D, 1:400, Minneapolis, MN, USA), and mouse anti Nestin (Abcam, 1:1000, Cambridge, UK).

### 2.7. The Tissue Clearing/Immunofluorescent Light-Sheet Imaging

*Tissue Clearing* [23]: After transcardial perfusion with 4% PFA, spinal-cord tissues were post-fixed in 4% PFA and then washed in PBS (both overnight at 4 °C). The dura was carefully and completely removed, as residual dura can trap bubbles that prevent effective light-sheet imaging. The spinal-cord tissue was cut into 1.0 cm–long segments, encompassing the injury site. Samples were incubated (on a rotating shaker at room temperature) in 50% Reagent I (diluted in ddH_2_O), with gentle shaking for 3 h, after which the solution was exchanged and samples were immersed in the same volume of fresh Reagent I for an additional 6–7 days until the sample became clear (changed with fresh Reagent I for every two days). After immunofluorescent labeling, samples were washed with PBS/NaN_3_ for 3 × 3 h at room temperature, while gently shaking, immersed in 50% Reagent II (10 mL per sample) for 3 h, after which the solution was exchanged and the samples were immersed in the same volume of fresh Reagent II for an additional 4–5 days (changed with fresh Reagent II for every two days).

*Reagent I:* 25 WT% Urea (Sigma V900119, Darmstadt, Germany), 25 WT% N, N, N′, N′-Tetrakis (2-hydroxypropyl) ethylenediamine (Quadrol, Sigma 122262, Darmstadt, Germany), 15 WT% Triton (VWR 0694-1L), and ddH_2_O.

*Reagent II:* 25 WT% Urea (Sigma V900119, Darmstadt, Germany), 50 WT% sucrose (Sigma S9378, Darmstadt, Germany), 10 WT% 2,2′,2″-Nitrilotriethanol (triethanolamine; Sigma 900257, Darmstadt, Germany), and ddH_2_O.

*Immunofluorescent labeling:* After the Reagent I clearing step, samples were washed in PBS/NaN_3_ solution for 3 × 3 h, and then incubated with 10% NGS/PBS blocking buffer for 24 h. Samples were then permeabilized in a solution with primary antibodies for 48 h. The samples were subjected to immunofluorescent labeling for several cell markers (glial fibrillary acidic protein (GFAP) and Neurofilament160 (NF160)). The primary antibodies were then washed in PBS/NaN_3_ (3 × 3 h), followed by incubated for 48 h with secondary antibodies. For nuclear staining, 0.1% DAPI was added in Reagent I during the whole immunostaining process. The samples were incubated in Reagent II plus ddH_2_O solution (NA = 1.45) and degassed before imaging. After imaging, the sample was washed with Reagent II again and immersed in Reagent II for storage.

*Light-sheet microscopic imaging:* Light-sheet Z.1 detection optics 5×/0.16; zoom, 0.8; laser lines, 405, 488, and 561 nm; ZEN 2012 (black edition).

### 2.8. Imaging Quantification and Statistical Analysis

The images that were obtained were processed and quantified with ImageJ software. A *t*-test, one-way ANOVA, and two-way ANOVA were used to assess statistical significance between independent experimental groups. Three-dimensional imaging reconstruction and surface panel image were processed with Imaris software.

### 2.9. BMS Scoring

Open-field locomotor activities were assessed by using the BMS locomotor rating scale [24] from 5 days after injury, weekly for 16 weeks post transplantation until animals were sacrificed. Briefly, mice were observed in the open-field for 1–2 min. Motor function of the hind-limbs was rated, recorded, and converted to a score according to the published scale.

### 2.10. Electrophysiology Analysis

Electrophysiological analysis was performed for each group at 7 weeks and 14 weeks post-injury, using Keypoint-II bichannel evoked potential/electromyography (Dantech). Uninjured mice with same age and gender were recorded as normal controls. All the animals were anesthetized by intramuscular (IM) injections of ketamine (20 mg/kg). For MEP recording, two stimulating electrodes were included: the positive electrode was placed on the skull surface of the motor area of the cerebral cortex (AP + 1.0, L/R ± 1.5, DV 0, mm from Bregma), 1 mm behind the Bregma and 1.5 mm on the left or right side from the midline; and the negative electrode was placed on the skull 0.5 cm lateral to the positive electrode. The recording electrode was inserted into the left or right gastrocnemius muscle of hind-limbs, with a depth of 1.5 mm. Moreover, the reference electrode was placed 2 cm away from the recording electrode, and the grounding line was placed in the middle of the stimulating electrode and recording electrode. A 0–10 mA single square wave (1 Hz) was applied to stimulate the motor area of the cerebral cortex through the skull with a duration of 0.2 ms. MEP was recorded at the gastrocnemius muscle of the hind-limb, and peak-to-peak amplitudes were calculated as amplitude values of MEP (Amp).

### 2.11. RNA Extraction and Quantitative Real-Time PCR Analysis

Total RNA was isolated by using RNAiso Plus reagent (TaKaRa, Shiga, Japan). The purified RNA was assessed by Nanodrop 2000 (Thermo Scientific, Waltham, MA, USA) to check the quality and quantity. PrimeScript™ RT reagent Kit with gDNA Eraser (Takara, Japan) and 1 μg of RNA was used to synthesize complementary DNA (cDNA). SYBR Premix EX Taq with ROX (TaKaRa, Japan) was used for performing the real-time PCR on a QuantStudio™ 5 System (Applied Biosystems, Waltham, MA, USA). The mRNA levels of target genes were normalized on the basis of GAPDH mRNA levels in each sample. The 2-ΔΔCT method was used to calculate gene expression. All primer sequences used in the experiment are listed in Supplementary Table S1.

### 2.12. Neurosphere Culture In Vitro

All mice were anesthetized and euthanized in accordance with Tongji University’s institutional guidelines. Brain and spinal-cord tissues were quickly removed from the body and placed into a 60 mm dish with cold HBSS (Gibco, Carlsbad, CA, USA). The subventricular zone (SVZ) of brain or spinal-cord specimens was further dissected under a stereomicroscope and then transferred to a 15 mL centrifuge tube. After washing the specimens three times with cold HBSS, tissues were digested with papain (Worthington, Lakewood, NJ, USA), at 37 °C, in a water bath, for 30 min. Cell suspension was filtered with a 40 μm cell strainer. After centrifuging at 300× *g* for 5 min, the cell pellet was resuspended with DMEM-F12 supplemented with 1 × B27 (Gibco, USA) in a tissue culture flask placed in a humidified incubator (37 °C, 5% CO_2_, 95% air). Then 20 ng/mL FGF-basic (Peprotech, Cranbury, NJ, USA) and 20 ng/mL EGF (Sigma, Darmstadt, Germany) were added every day to keep cells growing. Half of the medium was refreshed every 3 days. After 6 days, the floating neurospheres were harvested and incubated with 1 mL of Accutase (Gibco, USA) to be dissociated into single-cell suspensions for following experiments.

### 2.13. Cell Apoptosis Assay

NSCs were cultured with Bio-C for 72 h. The ANNEXIN V–FITC/PI Apoptosis Detection Kit (Zomanbio, Beijing, China) was used to evaluate the apoptosis of NSCs.

### 2.14. Cell Viability Assay

NSCs were cultured with or without Bio-C for 72 h. Cell viability was determined by using Cell Counting Kit-8 reagent (Dojindo Laboratories, Kumamoto, Japan).

### 2.15. NSC Spontaneous Differentiation In Vitro

We plated neurospheres onto PDL-coated (PDL 10 μg/mL in sterile distilled water; Sigma, Darmstadt, Germany) coverslips or cell culture plate after cultured with or without Bio-C for 72 h. After three days, bFGF/EGF was withdrawn. Cells were then cultured for another 7 days without growth factors for spontaneous differentiation.

### 2.16. RNA Sequencing

Total RNA was extracted from injured spinal-cord segments, separately, in 3 regions (rostral to the injury site, the Bio-C implanting site, caudal to the injury site) from C57 mice without or with SCI 14 weeks after injury, using TRIzol (Invitrogen, USA) according to the manufacturer’s instruction. Then 300–500 ng of qualified RNA was subjected to VAHTS^®^ Universal V8 RNA-Seq Library Prep Kit for Illumina (Vazyme NR605, Nanjing, China) for library preparation, followed by Illumina Nova-PE150 (USA) Sequencing. The quality of RNA and library was assessed by using an Agilent Bioanalyzer 2100 (Agilent Technologies, Santa Clara, CA, USA).

### 2.17. WGCNA (Weighted Gene Co-Expression Network Analysis)

A signed weighted correlation network was constructed by using 19 samples by first creating a matrix of pairwise correlations between all pairs of genes with annotation. The resulting Pearson correlation matrix was transformed into a matrix of connection strengths (e.g., an adjacency matrix), using a power of 18 [25]. Then the topological overlap was calculated to measure network interconnectedness [26]. For each dataset, we used average linkage hierarchical clustering to group genes on the basis of the topological overlap dissimilarity measure (topological overlap = 1) of their network connection strengths. Using a dynamic tree-cutting algorithm and merging threshold at 0.4, we identified 16 modules.

### 2.18. Transcriptomic Analysis

The corrplot R package was used to perform correlation analysis. Differentially expressed gene (DEG) analysis was performed by R package DESeq2. GO analysis was carried out with the clusterProfiler R package. A *p* ≤ 0.05 was considered to indicate significance.

### 2.19. Statistical Analysis

Data are presented as mean ± SEM. Statistical significance was assessed by unpaired two-tailed Student’s *t*-test. Student’s *t*-test was used to analyze data between two groups. All experiments were repeated at least three times. In all tests, *p* ≤ 0.05 was considered a statistically significant difference between the mean values.

## 3. Results

### 3.1. Construction and Properties of Bio-C

**Figure 1:**
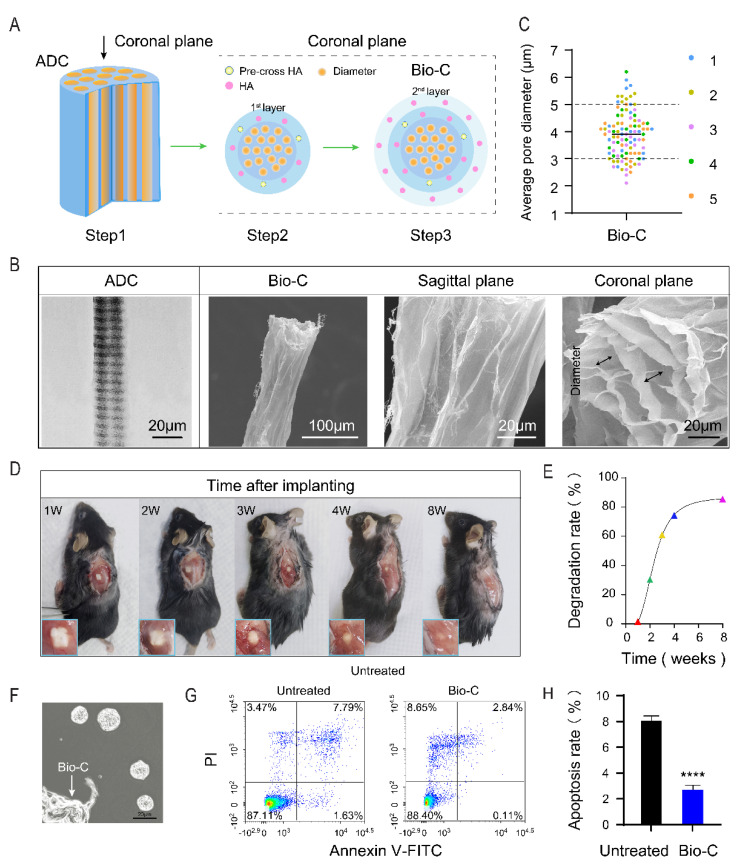
Characterization of Bio-C. (**A**) Schematic representation of Bio-C construction. (Step1) Bio-C constructed by the acid-desalted-collagen (ADC) and two outer HMW-HA layers. (Step 2) surface modification of purified ADC, using pre-crossed HMW-HA/and hydrated HMW sodium hyaluronate mixture to form first layer. (Step 3) Deposition of second layers of hydrated HMW sodium hyaluronate. (**B**) Electron micrographs of Bio-C scaffolds. TEM image showing ADC fiber at the core of Bio-C, maintaining collagen fibril structural integrity by periodic stripes with alternating light and dark shades (left). SEM image showing the morphology of Bio-C in sagittal plane and coronal plane (middle and right). (**C**) Statistical diagram showing the range of average pore diameters (arrow in **B**) from five batches of Bio-C. Over 70% of the samples carrying averaged pore sizes ranging between 3 and 5 μm. (**D**) Biodegradation and biocompatibility of Bio-C in vivo. Animals with Bio-C implants demonstrating different levels of material degradation from 1 week to 8 weeks. Enlarged images demonstrating mice with Bio-C implantations in the back muscle did not show signs of tissue rejection. (**E**) Degradation curve of Bio-C in vivo. The diagram revealed degradation kinetics of Bio-C from less than 10% at 1 week to over 85% at 8 weeks. (**F**) Morphology of neurospheres cultured in the presence of Bio-C. (**G**) NSCs apoptosis rates detected by PI/FITC-annexin V along combining with flow cytometric analysis. (**H**) Statistical diagram showing NSCs apoptosis rates with or without Bio-C. (**** *p* < 0.0001 significantly different from untreated control. Unpaired *t*-test. Data are presented as the mean ± SD.)

To build Bio-C, we chose ADC (collagen separated from bovine fascia through acid-washing, followed by desalting via dialysis) as the core of Bio-C (Figure 1A, Step 1). The triple-helix structural integrity was preserved, as observed under SEM (Figure 1B). ADC clearly maintained the structure of natural collagen (Figure 1B). Hydrated HMW sodium hyaluronate and pre-crossed HA mix, after “freeze and dry”, was used as the first layer of HA to modify ADC (Figure 1A, Step 2). Finally, hydrated HMW sodium hyaluronate was used as the second layer to coat ADC and give rise to Bio-C (Figure 1A, Step 3). The morphology of Bio-C under TEM (Figure 1B) displayed a microscopic surface in sagittal and coronal planes (Figure 1B). Robust porous structures with open channels suggest that Bio-C is probably beneficial for cell migration, as well as axonal elongation and outgrowth. The average pore diameter (bi-directional arrow bars in Figure 1B) of five batches of Bio-C were relatively consistent. More than 70% of the average pore diameters were in the range of 3–5 μm (Figure 1C), thus confirming the stability of Bio-C construction.

Bio-C was implanted into mouse back muscles and evaluated for its biocompatibility from 1 week to 8 weeks after surgical implantation. The implanted Bio-C was well tolerated without triggering immune rejections or foreign-object-induced body reactions, and it gradually degraded over time (Figure 1D). The size (volume) of the peripheral zone of the degraded Bio-C was observed and evaluated by ImageJ at each time point. Over 85% of implanted Bio-C was degraded at 8 weeks after implantation (Figure 1E). Therefore, Bio-C exhibited good biocompatibility and biodegradability in vivo. Furthermore, when Bio-C was placed in neural sphere cultures, NSCs displayed robust growth and reduced apoptosis, as judged by annexin-V binding assays (Figure 1F–H). Thus, Bio-C did not appear to have toxicity toward NSCs; instead, it appeared to be neurotrophic.

### 3.2. Bio-C-Enhanced Neural Regeneration and Functional Recovery in Mice after SCI

**Figure 2:**
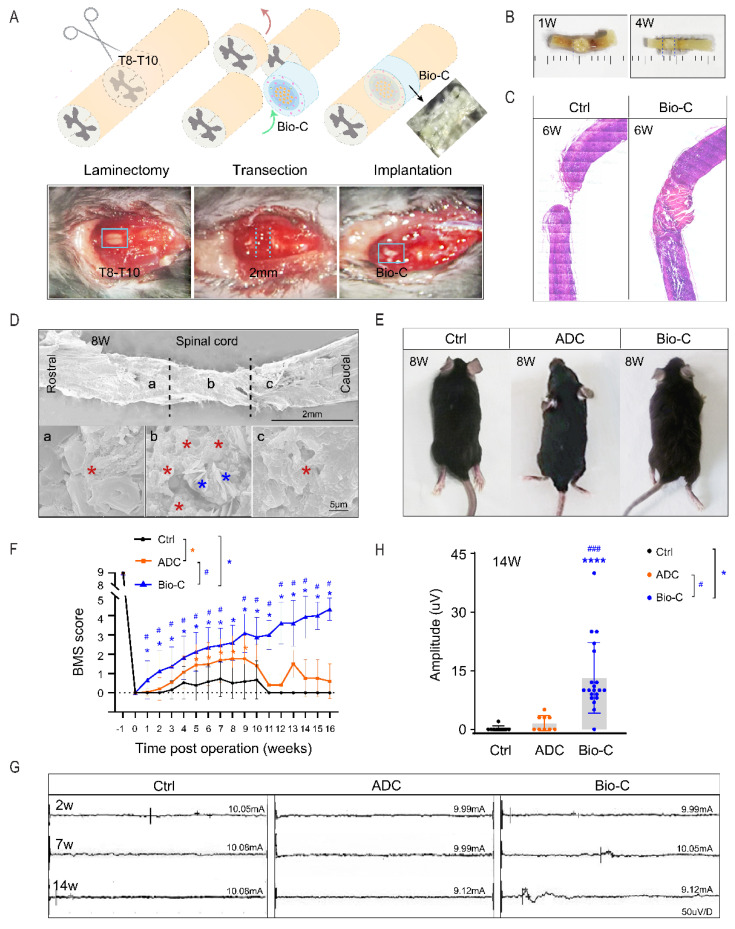
Bio-C promoted regeneration and functional recovery after complete SCI. (**A**) Schematic illustration of complete transection and segment removal SCI model (upper panel) and images of mice complete transection surgery (lower panels), in which a T8–T10 thoracic spine was open (laminectomy) and 2 mm–segment spinal-cord tissue was removed (transection), followed by immediate Bio-C or ADC implantation in the lesion area (implantation). (**B**) Freshly dissected spinal-cord tissue with Bio-C implants isolated in 1 week (left) and 4 weeks (right) after SCI. (**C**) Images of HE staining showing the difference of spinal-cord morphology with and without Bio-C implants at the 6 weeks after SCI. (**D**) Scanning electron micrographs showing spinal cord with Bio-C implants at 8 weeks after SCI. Red star indicates spinal-cord tissues, and blue star indicates Bio-C residue in tissue. (**E**) Different hind-limb recoveries in SCI (control), ADC, and Bio-C groups at 8 weeks after surgery. (**F**) Statistical diagram showing animal hind-limb motor-functional recovery after SCI in BMS open-field test (mean ± SEM; blue # indicates the difference between Bio-C and ADC mice; blue * indicates differences between Bio-C and SCI mice; orange * indicates differences between ADC and SCI mice; *# *p* < 0.05, *N* ≥ 165, *n* ≥ 3 unpaired Student’s *t*-test with comparisons of all three groups at each time point, up to 16 weeks). (**G**) Electrophysiological analysis of SCI mice with and without ADC and Bio-C implants at 2, 7, and 14 weeks after SCI. Schematic graph to show the MEP recording and representative MEP traces. Note that only the Bio-C group showed partial recovery at 14 weeks. (**H**) Statistics diagram showing significant MEP amplitude differences among SCI/control, ADC, and Bio-C groups at 14 weeks after SCI. (Mean ± SEM, *n* ≥ 3; **** *p* < 0.0001, and ### *p* < 0.001; unpaired Student’s *t*-test.) Both SCI and ADC groups had little MEP signals.

We examined the role of Bio-C in neural repair after SCI by implanting Bio-C in a completely transected with 2 mm-cord-segment-removal model of SCI (Figure 2A). The spinal-cord surgical procedure is illustrated in Figure 2A. In freshly isolated spinal-cord tissues with Bio-C, implants were clearly demarcated at 1 week after surgery and became more integrated at 4 weeks (Figure 2B). HE staining of SCI with or without Bio-C demonstrated robust fusion between spinal-cord tissues with Bio-C implants (Figure 2C). Some cells with nuclear staining could be detected within Bio-C implants 6 weeks after SCI (Figure 2C). SEM images showed that Bio-C and caudal/rostral lesion stumps had been closely fused together (Figure 2D) at 8 weeks after SCI. The fusion of Bio-C (blue star) and tissues (red star) was accompanied by the gradual degradation of Bio-C at the implant edge and the gradual penetration of new tissues into the implants (Figure 2D).

Locomotion performances of mice after SCI were photographed (Figure 2E and Supplementary Video S1) and assessed by using the Basso Mouse Scale (BMS) score in open-field test before injury and each week after injury for 16 weeks, in a double-blinded manner (Figure 2F). Animals without any implantation or ADC-implantation after SCI were considered to be in the control group. Bio-C mice demonstrated significantly better hind-limb recoveries compared to the ADC or SCI without treatment/control group from 1 week post-injury and onward, achieving a final average score of 4.6 out of 9 (non-injury level) (Figure 2F). In contrast, the ADC group had temporarily increased BMS scores from 1 week to 10 weeks post-injury, as compared to the SCI/control group, but scores remained lower than 2 (Figure 2F). The BMS scores of the ADC group continued to drop after 10 weeks, and they finally stayed at lower than 1 by 16 weeks post-injury. The SCI/control group had a score lower than 1 during the whole period. These results indicated that Bio-C was better than ADC in the perspective of stimulating better recoveries of locomotor activities in mice after SCI.

To further confirm motor-functional restorations, motor evoked potential (MEP) recording to assess the integrity of the motor pathway and functional recovery was carried out at 2, 7, and 14 weeks post-injury (Figure 2G). At 2 and 7 weeks, all three groups (including SCI/control, ADC, and Bio-C groups) had no responses; however, at 14 weeks, the amplitudes of MEP from the Bio-C group were significantly higher than those from ADC or SCI group, both of which had little, if any, visible signals.

Taken together, these data further supported the achievement of functional recovery after SCI that was elicited by Bio-C implants.

### 3.3. Transcriptomic Analysis Revealed Pathways Involved in Functional Recovery after SCI Elicited by Bio-C

**Figure 3:**
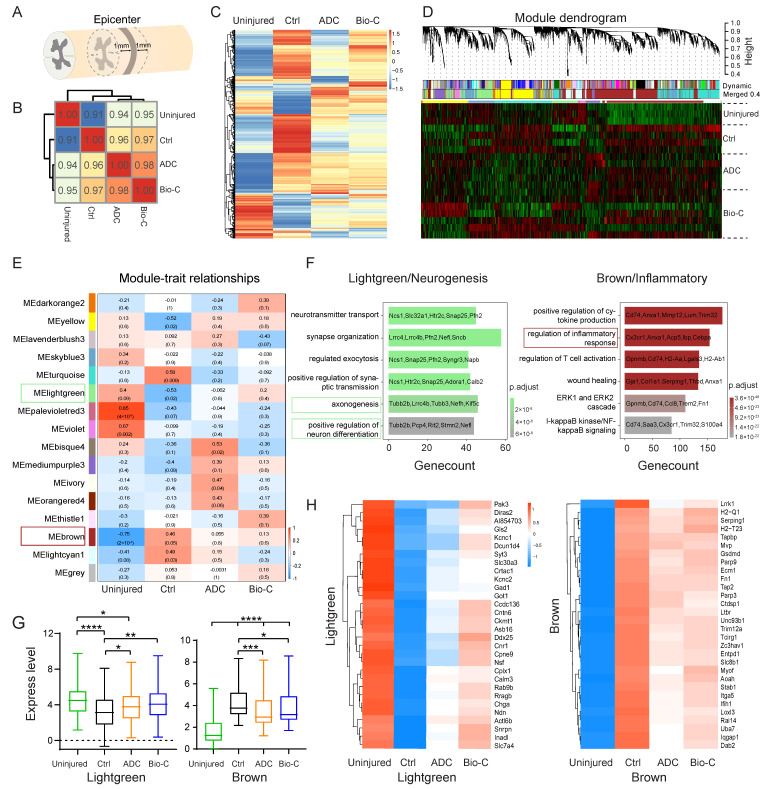
RNA-Seq analysis uncovered molecular mechanisms for better functional recovery in SCI mice with Bio-C implants. (**A**) Total RNA was collected from 2 mm segments of spinal cord surrounding the epicenter of the lesion. (**B**) Pairwise Pearson correlation amongst all sample groups (uninjured normal mice, injured control mice, and SCI mice with ADC or Bio-C implants). (**C**) Cluster thermogram of differentially expressed genes among sample groups (uninjured normal mice, injured control mice, and SCI mice with ADC or Bio-C implants). (**D**) WGCNA Dendritic Trees and Heatmap of significantly differentially expressed genes among sample groups before/after SCI and with the ADC or Bio-C implants. (**E**) Module–trait correlation analysis revealed dynamic changes of sixteen modules under different conditions. (**F**) GO analysis reveals the neurogenesis related “Light green” module and the inflammatory-related “Brown” module among 16 modules associated with the effect of Bio-C on SCI repair. (**G**) Box-plots of all hub genes in “Light green” module (87 hub genes) and “Brown” module (93 hub genes) (mean ± SEM, *n* ≥ 3; **** *p* < 0.0001, *** *p* < 0.001, ** *p* < 0.01, and * *p* < 0.05; unpaired Student’s *t*-test). (**H**) Heatmap of the top 30 hub genes according to “Light green” module and “Brown” module among uninjured normal mice, SCI control mice, and SCI mice with ADC and/or Bio-C implants.

To explore potential molecular mechanisms by which Bio-C promoted neural functional recovery after SCI, we carried out an extensive transcriptomic analysis, which was a more objective big-data-driven approach. Uninjured WT mice (number = 3), mice after SCI without treatment (number = 4), mice after SCI with ADC-implants (number = 4), and mice after SCI with Bio-C implants (number = 7) were sacrificed at 8 weeks post-surgery, and 2 mm–long segments of spinal-cord tissues at the lesion site (or the equivalent region of uninjured-spinal-cord tissues) were collected for total RNA extraction (Figure 3A).

The Pearson correlation coefficient of the averaged transcriptome/sample representing each group against other groups was calculated and is shown in Figure 3B. The SCI/control group is more different from the non-injury group as compared to the ADC or Bio-C group. The Bio-C group demonstrated the best correlation with the non-injury group, indicating that the regenerated spinal-cord tissues in the Bio-C group are most similar to normal (uninjured) states, indicative of the best therapeutic effect after SCI, as compared to the no-treatment or ADC groups (Figure 3B).

To understand which gene-expression program changed before and after SCI, we used unbiased Weighted Gene Co-Expression Network Analysis (WGCNA) on the complete dataset. We first performed differentially expressed genes (DEGs) analysis between uninjured, SCI-without treatment (control), ADC, and Bio-C groups, and a total of 10,106 genes were identified as DEGs (Figure 3C). We used those genes as input to conduct WGCNA.

A hierarchical cluster dendrogram of 18 samples identified co-expression gene modules (Figure 3D). Modules corresponding to branches were labeled with colors, as indicated by the color bands underneath the tree (Figure 3D). Initially, 56 gene clusters/modules were identified and were further merged into 16 modules after a 0.4 threshold merge (Figure 3D).

A cluster heatmap of the differentially expressed genes (DEG) among the sample groups (non-injury, SCI without treatment/control, ADC, and Bio-C) (*p* ≤ 0.05) was presented in Figure 3C. The figure clearly indicated that some genes were downregulated after SCI (comparisons between uninjured and injury/control groups) and were then elevated in the Bio-C group, and some followed the opposite trait. These genes were likely involved in neuronal functional recovery by Bio-C. Module–trait relationships revealed that almost all 16 modules had different expression patterns amongst the different groups (Figure 3E). A Gene Ontology (GO) analysis was performed to more thoroughly investigate signaling pathways involved in Bio-C elicited functional recovery. Among the 16 modules, the “Light green” module, which was involved in “positive regulation of neuronal differentiation”, “axonogenesis”, and “synapse organization”, as well as “neural transmitter transport”, was significantly downregulated after SCI and reversed expression with Bio-C implants following SCI (Figure 3E,F). In contrast, the “Brown” module, which was involved in “inflammatory response”, “wound healing”, and “positive regulation of cytokine production signaling pathway”, had elevated expression after SCI, and Bio-C implants also reversed their expression after SCI (Figure 3F). We plotted the averaged expression of all 87 hub genes belonging to the “Light green” module, as well as 93 hub genes belonging to the “Brown” module, amongst the four experimental groups (Figure 3G). In addition, we generated expression heatmaps of the top 30 hub genes of the “Light green” and “Brown” modules amongst the four experimental groups (Figure 3H). The expression patterns clearly indicated that, after SCI, “neural transmission” and “neuronal function” related genes were downregulated, but Bio-C reversed those changes, demonstrating its neural protective and pro-neural development functions. On the contrary, after SCI, “neural inflammation” was elevated, and Bio-C, as well as ADC, dampened the expression of inflammatory genes. The anti-correlation between the “Light green” and “Brown” modules also suggested that the two programs had interactions such that one negatively influenced the other, and vice versa (Figure 3H).

In summary, the role of Bio-C in neuronal functional recovery after SCI probably lies in the activation of neurogenesis and dampening of the inflammatory injury microenvironment.

### 3.4. Bio-C Improved NSCs Proliferation and Enhanced Differentiation of NSCs towards Neuronal Lineages

The activation of endogenous neural stem cells (NSCs) occurs during the pathological processes of SCI [27]. Transciptomic analysis suggested that Bio-C may also influence endogenous NSCs and their neuronal lineage differentiation. To test this hypothesis, we first investigated the role of Bio-C in cultured NSCs in vitro.

**Figure 4:**
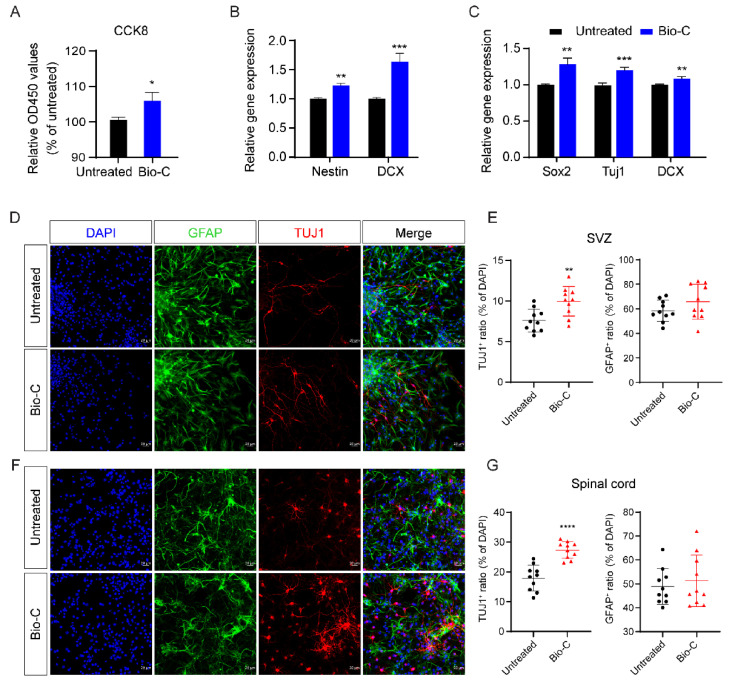
Bio-C improved NSCs growth and enhanced NSCs differentiation into neurons in vitro. (**A**) Viabilities of NSCs were detected by using “cell proliferation and cytotoxicity kit-8” (CCK8). (**B**) The mRNA expressions of Nestin (an undifferentiated NSC marker) and DCX (a newly differentiated neuronal marker) were measured by RT-qPCR. (**C**) The mRNA expressions of Sox2, Tuj1, and DCX (newly differentiated neuronal markers) were measured by RT-qPCR after 7 days of spontaneous differentiation culture. (**D**) Representative fields of GFAP and Tuj1 immunofluorescence staining of NSCs treated with Bio-C after 7 days of spontaneous differentiation. (**E**) Statistical analyses of (**D**) showing the ratios of Tuj1^+^/DAPI^+^ cells or GFAP^+^/DAPI^+^ in NSCs with or without Bio-C. (**F**) Representative fields of GFAP and Tuj1 immunofluorescence staining of sNSCs treated with Bio-C after 7 days of spontaneous differentiation. (**G**) Statistics analysis of (**F**), showing the ratios of Tuj1^+^/DAPI^+^ or GFAP^+^/DAPI^+^ cells in NSCs with or without Bio-C. NSCs were isolated from the SVZ of 4 weeks mice. sNSCs were isolated from the spinal cord of neonatal mice (postnatal day 1). (Note: **** *p* < 0.0001, *** *p* < 0.001, ** *p* < 0.01, and * *p* < 0.05; mean ± SEM, *n* ≥ 3; unpaired Student’s *t*-test.)

Bio-C was added to cultures of NSCs isolated from the forebrain subventricular zone (SVZ) surrounding the lateral ventricles of mice at postnatal 1 month. The CCK-8 analysis indicated that Bio-C increased NSC cell numbers (Figure 4A). Nestin was used here as a marker for undifferentiated NSCs; Sox2/Tuj1/DCX was used as a marker for newly differentiated neurons. When NSCs were maintained in proliferating conditions with Bio-C, the RT-PCR test showed that Nestin and DCX gene expressions were increased by Bio-C (Figure 4B), suggesting that Bio-C promoted NSC growth, as well as spontaneous differentiation into neurons. When NSCs were maintained in spontaneous differentiation conditions with Bio-C treatment, RT-PCR showed that gene expressions of Sox2/Tuj1/DCX were increased by Bio-C (Figure 4C), clear indicators for enhanced neurogenesis. To validate whether Bio-C also acted on spinal-cord NSCs in a similar way as SVZ NSCs, we cultivated spinal-cord neural stem cells (sNSCs) from postnatal day 1 mice (Supplementary Figure S1). Bio-C was added to NSCs and sNSCs separately in spontaneous differentiation conditions. The fluorescence intensities of Tuj1^+^ cells increased in both NSCs and sNSCs after Bio-C treatment (Figure 4D,F). The ratio of Tuj1^+^/DAPI^+^ cells increased from 18% up to 27% in sNSCs (spinal cord) and from 7.6% up to 10% in NSCs (SVZ) (Figure 4E,G). In contrast, the fluorescence intensities of GFAP^+^ cells did not change significantly in NSC or sNSC cultures after Bio-C treatment. These results supported the notion that Bio-C increased numbers of Nestin^+^ NSCs via reducing apoptosis of NSCs. Bio-C also either enhanced NSC differentiation into neurons or promoted the survival of newly differentiated neurons.

### 3.5. Bio-C Implants Promoted Neural Regeneration and Inhibited Microglia Activation after SCI

**Figure 5:**
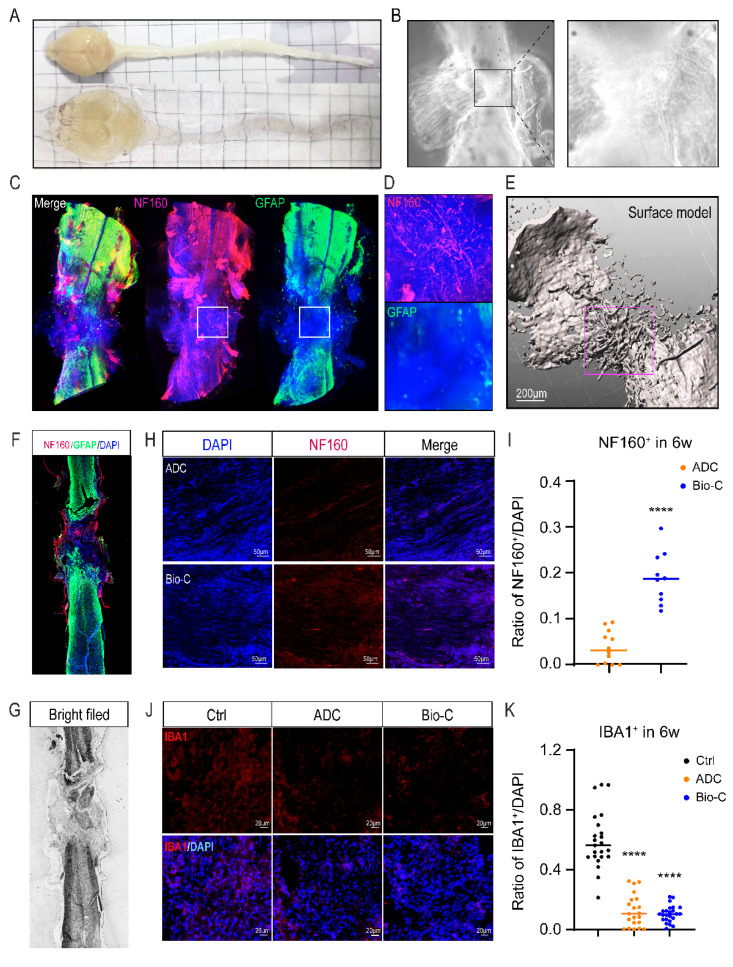
Bio-C increased NF160^+^ cell number and decreased IBA1^+^ cell number at 6 weeks after SCI. (**A**) Appearance of brain and spinal-cord tissues before and after CUBIC-clearing process. (**B**) Left: Bright field image showing spinal-cord tissue with Bio-C implants after clearing by using the CUBIC method. Right: a magnified image of the indicated area (the rectangle) in left. (**C**): The reconstituted 3D image showing distribution of NF160^+^ (red) and GFAP^+^ (green) cells in Bio-C implants at 6 weeks after SCI. (**D**) Magnified images of the indicated area (rectangles) in (**C**), showing plenty of NF160^+^ and DAPI^+^ but rarely GFAP^+^ distribution in the lesion/regeneration area. (**E**) Three-dimensional surface reconstruction of NF160^+^ fiber from (**B**), using Imaris software. (**F**) Representative fields of NF160 and GFAP immunofluorescence staining of tissue sections from spinal-cord segments with Bio-C implants. (**G**) Bright field image of (**F**). (**H**) Representative fields of NF160 immunofluorescence staining in lesion area with ADC and Bio-C implants. (**I**) Quantitative analyses of immunofluorescence showing the ratio of NF160^+^/DAPI^+^ cells in (**H**) (mean ± SEM, *n* ≥ 3, **** *p* < 0.0001, comparing Bio-C group to ADC group). (**J**) Representative fields of IBA1 immunofluorescence staining in lesion/regeneration area in SCI/control, and SCI with ADC or Bio-C implants. (**K**) Quantitative analyses of immunofluorescence, showing the ratio of NF160^+^/DAPI^+^ cells in (**J**) (mean ± SEM, *n* ≥ 3, **** *p* < 0.0001, comparing Bio-C group and ADC group to injured control group).

Raw Bio-C, initially without any cells inside (DAPI negative) (Supplementary Figure S2), became gradually fused with spinal-cord tissues after implantation into transected spinal cord, and endogenous cells could be found to infiltrate into the biomaterial at the lesion area. After clearing the spinal-cord tissues (Figure 5A,B) by using the CUBIC method, followed by immunofluorescent labeling with NF-160 (neuronal marker, red) and GFAP (astrocyte marker, green), light-sheet microscopy 3D imaging and video (Figure 5C–E, and Supplementary Video S2) were analyzed. The images demonstrated positive staining for NF160 and DAPI positive nuclei at the lesion site that were acellular at the beginning, i.e., right after SCI and Bio-C implantation, and this is indicative of tissue growth into the lesion/regeneration site (Figure 5C,D,F). GFAP^+^ astrocytes, on the other hand appeared to be repelled from entering the lesion and/or regeneration area (Figure 5C,D and Supplementary Figure S3). The morphology, density, and distribution of NF160^+^ cells can be observed by three-dimensional surface reconstruction via Imaris software (Figure 5C–E). This finding was consistent with the transcriptomic analysis indicating that neural functional recovery in Bio-C after SCI might be mediated through promoting the addition of neurons at the lesion site. To confirm this conclusion, immunostaining with NF160 and GFAP in tissue sections from spinal-cord segments encompassing the injury site (6 weeks after SCI) were photographed (Figure 5F,G) and cell numbers counted. There were less than 5% NF160^+^ neurons detected in the ADC implanted lesion area, whereas there were nearly 20% NF160^+^ neuronal cells in the Bio-C group (Figure 5H,I). Almost no neuronal staining could be detected across the lesion site in injured spinal cord without implants at 6 weeks after SCI, and this is indicative of no neurogenesis (Supplementary Figure S4).

Transcriptomic analyses also suggested that Bio-C was anti-inflammatory, and microglia were the major immune cells involved in inflammation within the CNS. To further confirm the immunomodulatory function of Bio-C, we performed immunohistochemical analyses by using IBA1 to label microglia at the lesion area after SCI. SCI-without-treatment/control, ADC, and Bio-C groups were analyzed at 6 weeks after SCI (Figure 5J,K). More than 50% of cells were IBA1 positive in the lesion area of the injury control group; however, less than 20% of the IBA1^+^ cells were detected in Bio-C and ADC groups (Figure 5J,K). These results suggested that both Bio-C and ADC could dampen inflammatory immune responses after SCI, which ought to be helpful for restoration of neural functional after SCI.

Taken together, Bio-C was neural protective and simultaneously anti-inflammatory at the lesion area after SCI and promoted functional recovery. ADC, on the other hand, was mainly anti-inflammatory. Considering the significant differences on BMS scores between Bio-C and ADC, both neurogenic/neural protective and anti-inflammatory functions of Bio-C appeared to be important to improve neural functional recovery after SCI.

In conclusion, we created a novel biodegradable material, Bio-C, with good biocompatibility and stable preparation process. Combining evaluations using in vitro NSCs cultures together with morphological, electrophysiological, objective transcriptomic, and behavioral analyses in vivo, we revealed that neural-tissue regeneration and functional recovery after SCI was achieved through the establishment of an anti-inflammatory, neurogenic, and neurotrophic microenvironment induced by Bio-C.

## 4. Discussion

### 4.1. Bio-C Promotes Neural Repair after SCI via Dual Functions

In this study, we constructed a new type of bioactive material composed of both collagen and HMW HA, referred to as Bio-C. Bio-C implants appeared to be able to promote SCI repair through eliciting neural protection and/or neurogenesis as well, as dampening inflammatory immune responses within an injured spinal cord. Previous reports indicated that biomaterial substrate stiffness affects the direction of differentiation of stem cells cultured on it [28]. HMW-HA was very close to that found in the ECM of CNS tissue; however, lower-molecular-weight HA scaffolds (<1 × 10^6^ Da) have difficulties in forming an intact porous structure and have no neural repair function [19,29]. This is why we used HMW-HA to construct Bio-C. Although ADC (collagen) alone could also elicit anti-inflammatory effects, similar to Bio-C, motor-functional recovery induced by ADC after SCI was quite limited (BMS scores under 2) and did not last long (less than 10 weeks). The reason why HMW-HA-modified Bio-C caused better neural functional recovery after SCI than ADC alone was probably that Bio-C was not only anti-inflammatory but also neurogenic. Previous research reported that the HMW form of HA could inhibit astrocyte proliferation in vitro and prevent astrogliosis in vivo after SCI [13]. Bio-C did not appear to have a strong impact on astrocytes in this study. Instead, it worked on neural stem cells (NSCs) and was robustly neurogenic. Bio-C has more than one function, in contrast to HMW-HA alone or collagen (ADC) alone. HMW-HA works on NSCs and/or perhaps astrocytes, and ADC, on anti-inflammation. Bio-C combines both properties of collagen and HA and is, therefore, more potent in SCI repair. Moreover, in our previous study, while NT3–chitosan has been shown to be very potent in neural repair, chitosan alone could not elicit neuro-regeneration, even though chitosan is also anti-inflammatory [6]. We speculate that Bio-C, if loaded with NT3, might elicit even more robust neural regeneration.

### 4.2. Powerful Transcriptomic Analysis

The SCI repair field has encountered quite some frustrations, in that research findings are difficult to be replicated from lab to lab and model to model. In the past 10 years, we have been advocating the usage of more objective big-data-based transcriptomic analyses of spinal-cord tissues, as these could help reveal the ongoing pathological processes in the spinal cord after injury. Severity of the injury and animal-to-animal variations could all be reflected by the transcriptome, such that the “black-box” of pathological events in injured or regenerating spinal cord could be exposed under the “sun”. Such an approach turned out to be very powerful [4,7,30]. Through this approach, we easily unveiled the potential mechanisms by which Bio-C elicited robust SCI repair, i.e., being neurogenic/neural protective and anti-inflammatory. This approach could also be utilized to compare results from lab to lab and model to model, and ultimately eradicate the aforementioned frustrations in the SCI research field.

### 4.3. Future Upgrades of Bio-C

In many studies, biomaterial scaffolds loaded with different types of cells, including NSCs or neurotrophic factors, have been fabricated and implanted into the spinal-cord lesion area to elicit neural regeneration [31,32,33,34]. Sometimes, ingrowth of new blood vessels [32], reduction of astrogliosis [35], and functional recovery [36] also occur. In our study, Bio-C displayed great biocompatibility with different NSCs from SVZ and spinal cord. The ability of Bio-C to promote neurogenesis from NSCs makes Bio-C potentially a good cell carrier for stem-cell-based therapy. Hypothetically, the construction of a Bio-C-based system carrying stem cells from different sources or neurotrophic factors, such as NT-3, might further enhance its ability to actualize better neural functional recovery after SCI in the future.

## 5. Conclusions

We constructed a new biodegradable material, Bio-C, by combining collagen with HMW-HA for the improvement of the injury microenvironment in the spinal cord to support the regeneration of neurons and suppression of inflammation; our material resulted in having good functional recovery after SCI. Although clinical treatment of SCI remains a constant challenge, Bio-C-based therapies could potentially offer effective treatment over conventional medicine for SCI in the near future.

Furthermore, Bio-C has quiet good biocompatibility and biodegradability, as well as highly stable and reproducible preparation processes, making it a new bioactive material with great application potentials. Due to its significant effect on neural regeneration and anti-inflammation, we expect that, in the future, Bio-C will be applied in the repair of various CNS injuries, without just being limited to spinal-cord-injury repair.

## Data Availability

The accession numbers for the sequencing raw data and process data in this paper are Genome Sequence Archive in BIG Data Center(GSA, Beijing Institute of Genomics, Chinese Academy of Science): PRJCA008562.

## Supplementary Materials

The following are available online at https://www.mdpi.com/article/10.3390/pharmaceutics14030596/s1, Video S1. Locomotor activities of C57 mice at 8 weeks after SCI. Video S2. Three dimensional reconstruction (3D) of spinal cord specimen with Bio-C implants at 6 weeks after SCI surgery under lightsheet microscope. Figure S1. Characterization of neonatal spinal cord NSCs. Figure S2. Original Bio-C without any cells inside. Figure S3. Distribution of GFAP positive cells in injured mouse spinal cord with ADC or Bio-C implants after SCI. Figure S4. Distribution of MAP2 positive cells in injured mouse spinal cord after SCI.
