# Supplementary material for: Bio-C (Modified Hyaluronic Acid-Coated-Collagen Tube) Implants Enable Functional Recovery after Complete Spinal Cord Injury
Source: Pharmaceutics. 2022 Mar 9;14(3):596. doi: 10.3390/pharmaceutics14030596 (PMC8954105; doi:10.3390/pharmaceutics14030596)
Supplement: Supplementary file 1 [file pharmaceutics-14-00596-s001.zip › supplimentary Legend_final.pdf]

Supplementary Materials

Supplementary Video 1. Locomotor activities of C57 mice at 8weeks after SCI.

Animal with locomotion performance was recorded by Leica camera. Different performance of motor functional recovery were displayed in SCI mice without implants, SCI mice with ADC or Bio-C implants at 8weeks after SCI. Injured mice without implants showing rarely hindlimb recovery after 8 weeks (BMS scores in 0), SCI mice with ADC implants showing a little hindlimb recovery with slight hindlimb lifting and twitching (BMS scores in 2), SCI animal with Bio-C implants showing much better hindlimb recovery with stable hindlimb lifting and movement (BMS scores in 4).

Supplementary Video 2. Three dimensional reconstruction (3D) of spinal cord specimen with Bio-C implants at 6 weeks after SCI surgery under lightsheet microscope.

The spinal cord tissue after CUBIC clearing was imaged under a 5× objective (NA-1.45) using Lightsheet Z1 microscopy (Zeiss). Images were used for reconstruction. While the reconstruction, 3D rendering and video were generated on ARIVIS Vision 4D software. Immunostaining of NF160 with AlexaFluor 594 (red), GFAP with AlexaFluor 488 (green), DAPI for nuclei (blue).

Supplementary Figure 1. Characterization of neonatal spinal cord NSCs.

NSCs derived from neonatal mouse spinal cord were cultured *in vitro*. Spinal cord neural stem cell (sNSCs) colonies are positive for NSC markers Sox2 and Nestin.

Supplementary Figure 2. Original Bio-C without any cells inside.

DAPI staining showed no cells exit in freshly preparedBio-C.

Supplementary Figure 3. Distribution of GFAP positive cells in injured mouse spinal cord with ADC or Bio-C implants after SCI.

Representative images of GFAP immunofluorescence staining in spinal cord lesion area at 6 weeks after SCI. GFAP positive cells were located on both rostral and causal sides of the lesion area instead of the lesion core in injured mice with ADC implants or with Bio-C implants.

Supplementary Figure 4. Distribution of MAP2 positive cells in injured mouse spinal cord after SCI.

Representative images of MAP2 immunofluorescence staining in spinal cord lesion area at 6 weeks after SCI. Almost no MAP2 positive cells was found across the lesion site (beyond the white line in the figure) in the injured control mice without implants, indicative of no neural regeneration.

Supplementary Table 1. Primer sequences used for real-time quantitative reverse transcriptase polymerase chain reaction (qRT-PCR).

Target gene	Forward primer (5'--3')	Reverse primer (5'--3')
Gapdh	TGTGTCCGTCGTGGATCTGA	CCTGCTTCACCACCTTCTTGA
Gfap	ACCATTCCTGTACAGACTTTCTCC	AGTCTTTACCACGATGTTCCTCTT
Tubb3 (Tuj1)	CAACTATGTGGGGGACTCGG	TGGCTCTGGGCACATACTTG
Nes (Nestin)	GCAGAGAAGACAGTGAGGCAGATG	GGAGGCAGGAGACTTCAGGTAGAG
Dcx	CAGTCAGCTCTCAACACCTAAG	CATCTTTCACATGGAATCGCC
Sox2	ATGGACAGCTACGCGCAC	CGAGCCGTTCATGTAGGTCTG
