# Supplementary figures and images for: Bio-C (Modified Hyaluronic Acid-Coated-Collagen Tube) Implants Enable Functional Recovery after Complete Spinal Cord Injury
Source: Pharmaceutics. 2022 Mar 9;14(3):596. doi: 10.3390/pharmaceutics14030596 (PMC8954105; doi:10.3390/pharmaceutics14030596)

S1

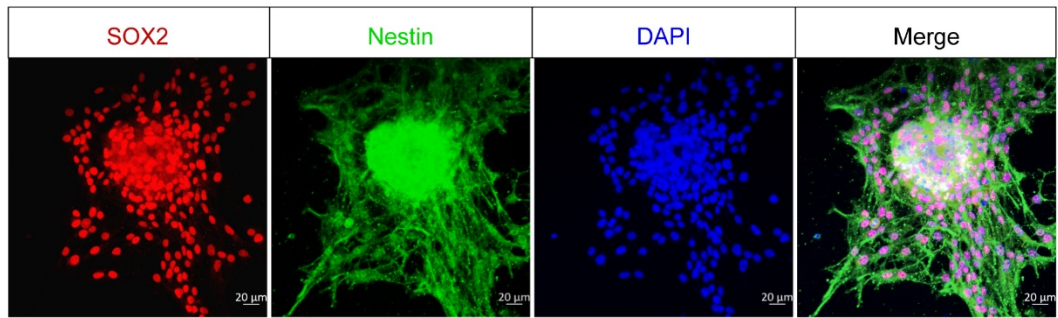

Supplement: Supplementary file 1 [file pharmaceutics-14-00596-s001.zip › Supplementary figure S1.pdf]

S2

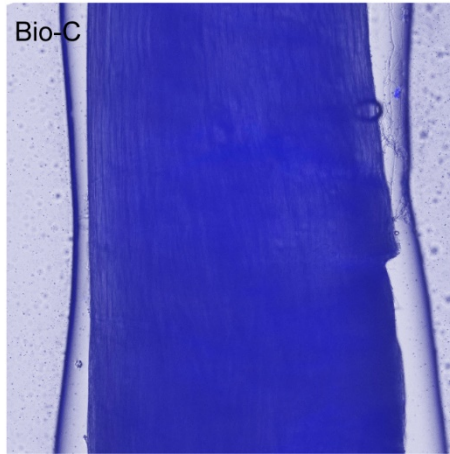

Supplement: Supplementary file 1 [file pharmaceutics-14-00596-s001.zip › Supplementary figure S2.pdf]

S3

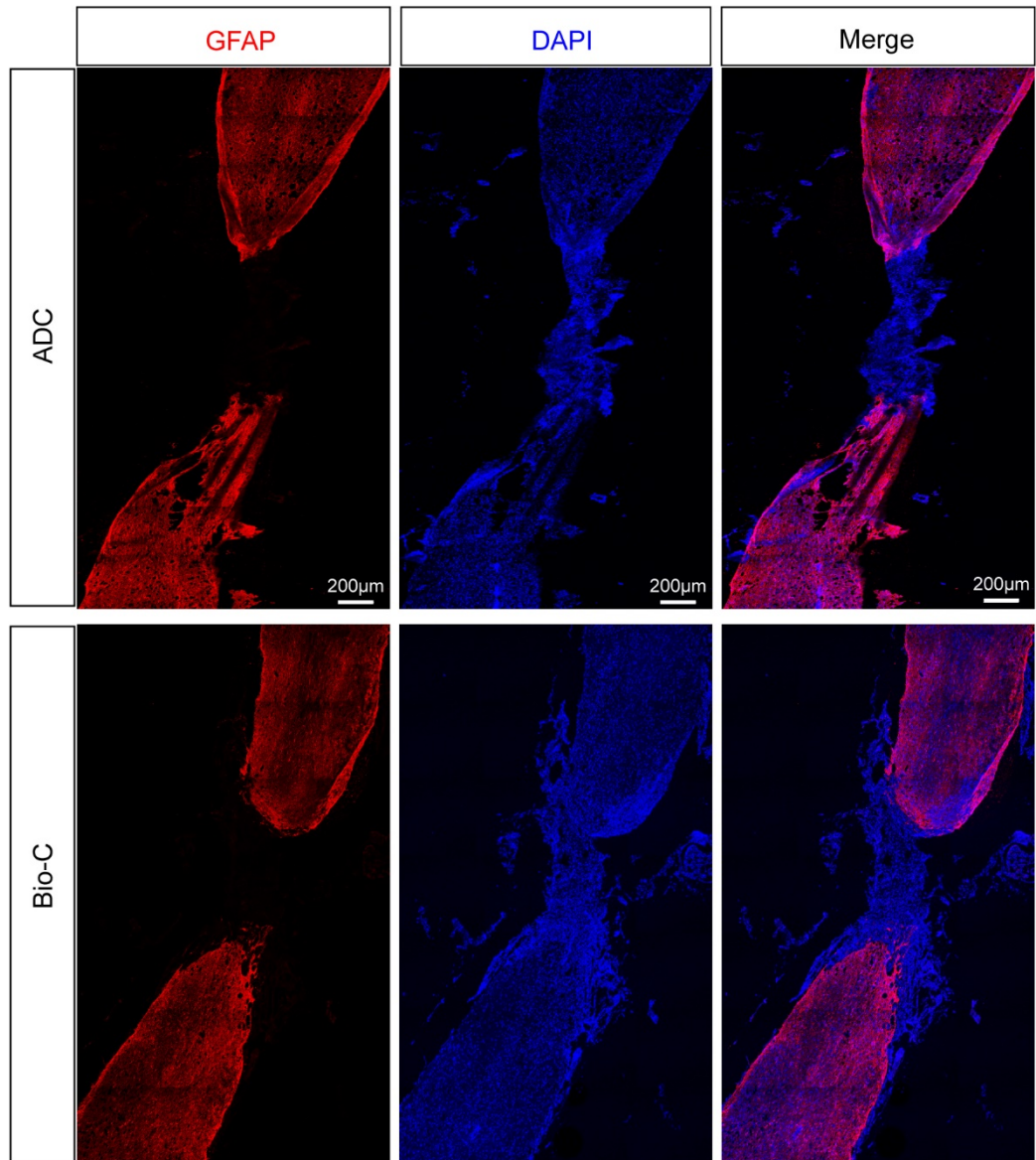

Supplement: Supplementary file 1 [file pharmaceutics-14-00596-s001.zip › Supplementary figure S3.pdf]

S4

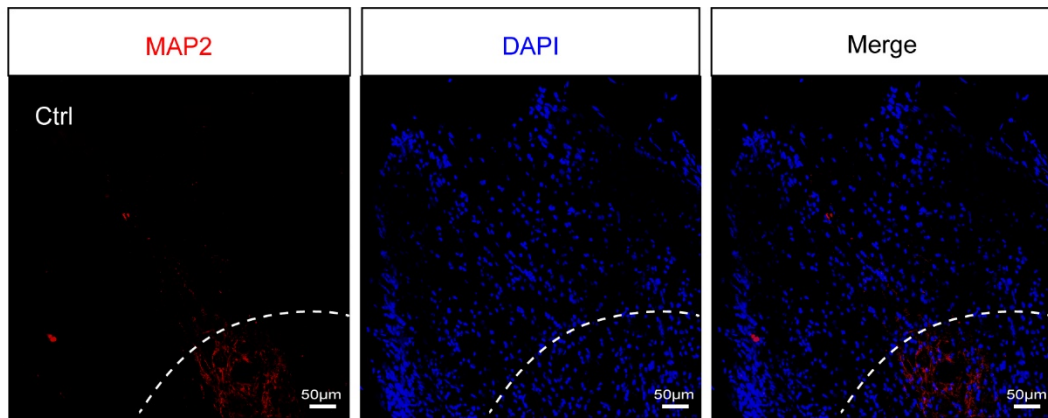

Supplement: Supplementary file 1 [file pharmaceutics-14-00596-s001.zip › Supplementary figure S4.pdf]
